# Metabolomic Alterations of Volatile Organic Compounds and Bile Acids as Biomarkers of Microbial Shifts in a Murine Model of Short Bowel Syndrome

**DOI:** 10.3390/nu15234949

**Published:** 2023-11-29

**Authors:** Vanessa Wolfschluckner, Beate Obermüller, Angela Horvath, Giovanny Rodriguez-Blanco, Patricia Fuchs, Wolfram Miekisch, Barbara Mittl, Christina Flucher, Holger Till, Georg Singer

**Affiliations:** 1 Department of Paediatric and Adolescent Surgery, Medical University of Graz, 8036 Graz, Austria; vanessa.wolfschluckner@medunigraz.at (V.W.); barbara.mittl@medunigraz.at (B.M.); christina.flucher@medunigraz.at (C.F.); holger.till@medunigraz.at (H.T.); georg.singer@medunigraz.at (G.S.); 2 Division of Gastroenterology and Hepatology, Medical University of Graz, 8036 Graz, Austria; angela.horvath@medunigraz.at; 3 Clinical Institute of Medical and Chemical Laboratory Diagnostics, Medical University of Graz, 8036 Graz, Austria; g.blanco@medunigraz.at; 4 Department of Anaesthesiology, Intensive Care and Pain Therapy, Rostock University Medical Center, 18057 Rostock, Germany; patricia.fuchs@uni-rostock.de (P.F.); wolfram.miekisch@uni-rostock.de (W.M.)

**Keywords:** bowel resection, intestinal segments, 16S rRNA gene sequencing, microbiome, mice

## Abstract

Pediatric short bowel syndrome (SBS) is a rare condition characterized by a massive loss of the small intestine, leading to the inability to meet nutritional requirements without the use of parenteral or enteral supplementation. SBS causes profound alterations in the intestinal microbiome and metabolome. The aim of this study was a detailed assessment of the intestinal microbiome and metabolome in a murine model of SBS. We performed a 60% proximal small bowel resection versus a sham operation in C57BL/6 mice. Four weeks postoperatively, the microbial communities of different intestinal segments (jejunum, ileum, colon) and stool were assessed by 16S rRNA gene sequencing. Bile acids in serum and stool and volatile organic compounds (VOCs) in the fecal headspace were assessed using LC-MS and GC-MS techniques. The α-diversity of the different intestinal segments did not significantly differ between the two groups. β-diversity significantly differed between sham and SBS mice. While in the jejunum, *Faecalibaculum* was significantly increased in SBS animals, a significant reduction in *Lactobacillus* and *Sporosarcina* was detected in the ileum of SBS mice. In the colon of SBS mice, a significant decrease in *Ruminococcaceae* and a significant increase in Proteobacteria such as *Faecalibaculum* and *Escherichia-Shigella* were found. Serum levels of deoxycholic, taurocholic and taurochenodeoxycholic acids were significantly higher in the SBS group. Of the 29 VOCs tested, hexane, isoflurane and pentane were significantly higher in the SBS group, and pyrrole was significantly lower. We were able to show that SBS causes shifts in the murine intestinal microbiome and metabolome including serum BAs and fecal VOCs.

## 1. Introduction

Pediatric short bowel syndrome (SBS) represents a rare condition characterized by a massive loss of the small intestine, leading to the inability to meet nutritional requirements without the use of parenteral or enteral supplementation [1,2]. The North American Society for Pediatric Gastroenterology, Hepatology and Nutrition (NASPGHAN) has defined SBS as “the need for parenteral nutrition for >60 days after intestinal resection or a bowel length of less than 25% of expected” [3].

Amongst other outcomes, SBS leads to excessive fluid and electrolyte losses and results in substantial malabsorption of macronutrients, vitamins and minerals [4]. As a consequence, it impacts the ability to gain weight and achieve normal growth and development and therefore remains a persistent medical challenge severely impacting both patients and their families [5].

Furthermore, SBS patients often suffer from small intestinal bacterial overgrowth (SIBO) leading to symptoms such as abdominal distension and pain, bloating, nausea, intolerance of enteral nutrition, diarrhea, dehydration, weight loss and metabolic acidosis [6]. Moreover, the incidence of sepsis remains high and is the most common cause of readmission of SBS patients, increasing the length of hospitalization as well as the cost of care [7,8].

It is assumed that the occurrence of SIBO is promoted by increased bowel caliber and reduced peristalsis of the remaining intestine causing profound alterations in the gastrointestinal microbiome [9]. These microbial alterations have already been shown in a variety of different clinical and experimental studies [10,11,12,13].

SBS also leads to disruptions of the enterohepatic circulation resulting in disturbed bile acid (BA) metabolism and a preference for microorganisms tolerant to BAs, thereby fueling intestinal dysbiosis. Previously published studies suggest that due to the BA deficiency resulting from the interruption of the enterohepatic circulation in SBS, there is likely an increased hepatic synthesis and uptake of BAs [14]. Furthermore, it is suggested that enhanced bacterial deconjugation of BAs in the intestine leads to increased passive absorption and elevated serum levels [15].

Volatile organic compounds (VOCs) originate from biochemical reactions triggered by processes such as inflammation, oxidative stress and cellular demise. Other substances are taken up from the environment or previous load (e.g., drugs, nutrition). Certain compounds exhibit significant variation based on pathophysiological processes and metabolic distinctions, potentially serving as non-invasive biomarkers of specific states of health or disease [16,17]. Studies have demonstrated alterations in VOCs in various conditions, including irritable bowel syndrome [18,19]. However, alterations in VOCs in the setting of SBS are still poorly understood. 

The aim of this study was therefore a detailed assessment of the metabolome and microbiome of a murine model of SBS. We sought to analyze alterations in the microbial community of the different intestinal segments as well as alterations in the metabolome (BAs, VOCs and inflammatory parameters) in a mouse model of a 60% proximal small bowel resection.

## 2. Materials and Methods

### 2.1. Animals

Following approval of the veterinary board (2020-0.380.477), 22 male C57BL/6 mice were ordered from Envigo Lab (Indianapolis, IN, USA) at the age of 12 weeks. The mice were housed individually under specific-pathogen-free (SPF) conditions and in standardized individually ventilated cages (IVC) at the Center for Biomedical Research (BMF) at the Medical University of Graz. They were given free access to water and solid food at any time and were exposed to a 12 h light and dark cycle. Daily weight and health checks were carried out at the same time. After an adjustment phase of one week, the solid food was switched to a liquid diet (AIN93G Water soluble, ssniff, Soest, Germany) one week preoperatively, and an autoclaved piece of wood was provided for gnawing in the cage.

### 2.2. Operation Method

One hour before the planned operation, the animals received intraperitoneal analgesia with buprenorphine 0.1 mg/kg body weight. After inhalation anesthesia was induced with isoflurane (2–5 Vol%) and the abdomen was shaved, the surgical site was disinfected and a midline laparotomy was performed. The operation was performed using a microscope at 8-fold magnification. 

After dissecting the muscular tissue and opening the peritoneum, both the cecum and the entire small intestine were exteriorized. Care was taken to keep the intestines moist at all times. 

In the first group (*n* = 14 animals), SBS was induced by performing a 60% proximal small bowel resection, 2 cm distal to the ligament of Treitz and 10 cm proximal to the ileocecal valve, as previously published [20]. In the second group (*n* = 8), a sham operation was performed involving a transection of the small intestine approximately 12 cm proximal to the ileocecal valve, followed by reanastomosis without resection. The anastomosis was performed using 10/0 monofilament interrupted sutures, and the abdomen was closed in two layers using absorbable suture material.

After a short recovery phase, the animals were again provided with free access to water and liquid chow.

### 2.3. Euthanasia and Sample Collection

The mice were weighed on a daily basis, and the weight changes were recorded. At the end of a 4-week observation period, the mice were orally administered 500 mg/kg of a FITC dextran solution (50 mg/mL solution in PBS buffer) through gavage, 16 h prior to euthanasia, to assess intestinal permeability. Subsequently, each mouse received a subcutaneous injection of 400 μL isotonic saline solution 1 h before euthanasia. At the same time, fecal pellets were collected for BA, microbiome and VOC analysis. Deep isoflurane anesthesia was induced to ensure the mice were in a profound state of unconsciousness. The hearts of the animals were then punctured to extract as much blood as possible. To confirm death, craniocervical dislocation was performed.

The collected blood was centrifuged, and the resulting serum was separated, distributed into multiple Eppendorf tubes and frozen at −80 °C for future investigations. Organs including the liver, spleen, kidneys, adipose tissue and pancreas were dissected and weighed [21]. Furthermore, stool contents of the colon, ileum and jejunum were collected for microbiome analysis. Cecal contents were taken for analysis of SCFAs.

### 2.4. Gut Permeability

For FITC analysis, the serum samples were shielded from light and kept at 6 °C until measurement. Within 8 h of photometric collection, FITC-dextran serum levels were assessed using a FLUOstar Omega instrument (BMG LABTECH, Ortenberg, Germany) at an absorption wavelength of 485 nm and an emission wavelength of 535 nm [22]. Standard curves were established following the manufacturer’s protocol.

### 2.5. Cytokines and Chemokines

A customized antibody-based, magnetic bead mouse ProcartaPlex^TM^ (ThermoFisher Scientific, Waltham, MA, USA) targeting ENA-78, G-CSF, GM-CSF, IFN beta, IFN gamma, IL-1 beta, IL-10, IL-17F, IL-2, IL-2R, IL-6, M-CSF, MCP-1, MCP-5, MIP-1 alpha, MIP-1 beta, TNF alpha and CRP was designed and executed as described in the manufacturer’s instruction booklet. Briefly, all needed standards were combined and diluted as specified. After adding the beads to the 96-well plate, 25 µL samples of standards or serum were placed in the set wells and incubated for 2 h at room temperature with shaking at 600 rpm. After the wells were cleaned, 25 µL detection antibody was added and incubated for 30 min at room temperature with shaking. Following several washing steps, 50 µL Streptavidin-PE was pipetted in and incubated again for 30 min at room temperature with soft shaking. Again, the plate was washed, and 120 µL reading buffer was added to resuspend the beads. After an incubation time of 5 min at room temperature with shaking, the plate was analyzed with a BioPlex-200 (Bio-Rad, Hercules, CA, USA). The same procedure was performed for the CRP magnetic bead assay, but the serum was diluted 1:1000 as recommended.

### 2.6. Tight Junction Proteins

The analyses of the claudin protein family and the alpha-amino acid citrulline were performed with colorimetric assay kits (claudin 2: E03C0983; claudin 4: E03C0970, occludin: E03O0009; citrulline: E03C0043; Blue Gene). Samples were diluted 1:7 for the claudin protein family and 1:35 for citrulline. All assays were handled according to the manufacturer’s instructions. Briefly, 100 µL of all standards after the recommended dilution series and all diluted sera were added to the 96-well plate according to the loading scheme. Then, 50 µL conjugate was pipetted in every well except the blank and incubated for 1 h at 37 °C. After several washing steps, 50 µL of Substrate A and 50 µL of Substrate B were added to all wells and incubated again for 20 min at 37 °C. Afterward, 50 µL of stop solution was placed in each well, and the OD was measured at 450 nm immediately.

### 2.7. Short-Chain Fatty Acids

Cecal content was homogenized in distilled water, followed by the extraction of two 100 µL samples. The first sample was dried, and its mass was measured to standardize the second aliquot. The second aliquot was processed as outlined below.

To this aliquot, 100 µL of internal standards including acetic acid-d4, propionic acid-d6, butyric acid-d8, valeric acid-d9 and hexanoic acid-d3 was added, resulting in a final concentration of 100 µM. After 30 min of agitation, 100 µL of the resulting supernatant was transferred to a glass Pyrex tube. Thereafter, 10 µL of 3% phosphoric acid and 1 mL of methyl tert-butyl ether (MTBE) were introduced. The mixture was shaken for 10 min and then centrifuged in a laboratory centrifuge at 2500 rpm. The upper phase was moved to a GC-MS vial.

For injection into the gas chromatography–mass spectrometry (GC-MS) system, a 1 µL volume was utilized in splitless mode at an injector temperature of 250 °C. The GC oven was furnished with a 1 DB-WAX UI GC-column (30 m × 0.25 mm ID × 0.25 µm film). The initial oven temperature of 40 °C was sustained for 2 min, followed by a ramp to 150 °C at a rate of 15 °C per minute. Subsequently, there was an increase of 5 °C per minute to 170 °C, succeeded by a rise of 20 °C per minute up to 250 °C, where the temperature was maintained for 5 min. Helium functioned as the carrier gas at a flow rate of 1.3 mL/min. The transfer line temperature was established at 280 °C.

The mass spectrometer was operated in selected ion monitoring (SIM) mode, targeting specific *m*/*z* values (60, 63, 73, 74, 76, 79 and 80) at their respective retention times. Quantification of acetic (C2), propionic (C3), butyric (C4), valeric (C5) and hexanoic (C6) acids was conducted through single-point calibration in comparison to their respective internal standards.

### 2.8. Bile Acids in Serum and Fecal Samples

After introducing internal standards (d4-DCA, d4-LCA, d4-GLCA, d4-GCDCA and d4-TDCA, each at a concentration of 0.2 nmol), samples (10 μL) were vigorously mixed for 1 min. To facilitate the removal of proteins, 400 μL of acetonitrile was added. The mixture was vigorously mixed again and then subjected to centrifugation at 3200× *g* for 12 min at room temperature. The supernatant was then carefully removed and subsequently evaporated under a flow of nitrogen. The dried samples were reconstituted using 100 μL of mobile phase B and subsequently transferred into vials suitable for autosampling.

BAs from stool samples were extracted according to previous publications [23,24]. Snap-frozen stool samples (10 mg) were incubated with NaOH (0.1 M, 2 mL) for 60 min at 60 °C. After 4 mL of aqua dest. was added, samples were homogenized, and proteins were denatured by adding 80% *v*/*v* of acetonitrile for 20 min at room temperature. Internal standards were added as indicated above. Proteins were removed by 20 min of centrifugation at 20,000× *g*. The supernatant was dried at 50 °C under nitrogen flow, and the pellet was re-dissolved in 4 mL of ammonium acetate and purified using C18 reversed-phase SPE cartridges. To remove hydrophilic material, 20 mL of aqua dest. was used, whereas lipophilic components were removed with 10 mL of hexane. Finally, BAs were eluted with 2 mL of methanol. 

BAs were analyzed by liquid chromatography–high-resolution mass spectrometry (LC-HR-MS). Chromatography of 10 µL of each sample was performed using a Nucleoshell C18 reversed-phase column (Macherey-Nagel, Düren, Germany) for human BAs. Separation was performed using aqua dest. with 1.2% *v*/*v* formic acid and 0.38% w/v ammonium acetate, and elution was carried out using acetonitrile with 1.3% *v*/*v* formic acid and 0.38% ammonium acetate. Analysis was performed on a Triple Quadrupole mass spectrometer 6500 (Sciex, Framingham, MA, USA) with an ESI ion source in negative ionization mode. Two different acquisition methods were used: A 20 min and fully validated method for the analysis of primary, secondary and conjugated BAs was used for the quantitation in serum and stool. An extended 60 min method, including murine-specific BAs and other isobars, was used for the qualitative analyses. The limit of quantitation of the mass spectrometer was 0.001 µmol/L for all BA species. Any value below this threshold was not quantitated and thus excluded from statistical analyses. For the qualitative analyses, statistical comparisons were performed using normalized peak areas.

### 2.9. Fecal Volatile Organic Compounds

Samples were weighed and placed in 20 mL gas-tight glass vials (Gerstel GmbH, Mülheim an der Ruhr, Germany) and then stored at a temperature of 6 °C. Concurrently, room air samples were gathered at the same time points to account for potential contamination. Without delay, all samples were dispatched to the collaborating partner via overnight express for gas chromatography/mass spectrometry (GC/MS) analysis. VOCs were analyzed in the headspace of the samples, following established methodologies [25,26,27]. VOCs were preconcentrated using a commercially available solid-phase microextraction (SPME) fiber (carboxen/polymethylsiloxane, Supelco, Bellefonte, PA, USA) which was thermally desorbed in the GC injector and analyzed by GC/MS. An Agilent 7890 A gas chromatograph (GC) coupled with an Agilent 5975 C inert XL mass-selective detector (MSD) was applied to separate and identify VOCs desorbed from the SPME device.

For identification, detected volatile marker compounds were provisionally matched using a mass spectral library (National Institute of Standards and Technology 2005; NIST 2005, Gatesburg, PA, USA) and retention time comparisons. The results were adjusted based on the weight of the stool. In instances where the average of the room air samples surpassed 30% of the mean of the headspace samples, potential contamination was acknowledged, and the respective compound was omitted from subsequent analysis. The area responses (area under the curve (AUC)) of a selected m/q ratio at a defined retention time for each substance were recorded, integrated and used for group comparison.

### 2.10. Microbiome

For the isolation of total DNA from fecal samples, the QIAsymphony DSP Virus/pathogen Mini Kit; 192 rxn (937036) (Qiagen, Venlo, The Netherlands) was employed, following established protocols [28]. Briefly, a single stool pellet was diluted in 500 μL of PBS, and 100 μL of pre-diluted stool suspension plus 600 μL of Qiagen ATL Buffer were subjected to bead beating using Magna Lyser Tubes (Roche, Mannheim, Germany) and a Magna Lyser instrument (Roche, Mannheim, Germany). The bead beating was performed twice at 6500 rpm for 30 s each time. Subsequently, enzymatic lysis was conducted using 35 μL of lysozyme (100 ng/mL) at 37 °C for 30 min, followed by 70 μL of proteinase K (Oiagen-Kit, 20 mg/mL) at 68 °C for 1 h. The samples were heat-inactivated at 95 °C for 10 min, and the resultant total DNA was purified using a QIAsymphony SP (Qiagen) instrument, following the manufacturer’s instructions. The eluted total DNA was collected in 110 μL of elution buffer and stored at −20 °C until analysis.

For the 16S PCR, 2 μL of the total DNA served as the template in a 25 μL PCR reaction, utilizing the FastStart^TM^ High Fidelity PCR-System (Sigma, Darmstadt, Germany) and the specified primers 515F (5′-GTGYCAGCMGCCGCGGTAA-3′) and 806R (5′-GGACTACNVGGGTWTCTAAT-3′) for 30 cycles, performed in triplicate. The triplicate PCR products were pooled, normalized, indexed and purified in line with established protocols [28]. The final pool was subjected to sequencing on an Illumina MiSeq desktop sequencer using 9 pM and v3 600-cycle chemistry.

For data analysis, the MiSeq paired-end FASTQ reads totaling 2,479,083 were utilized. The DADA2 pipeline was employed to model and correct Illumina-sequenced amplicon errors for quality filtering, employing standard settings for denoising, dereplication, merging, and chimera checking, implemented within the QIIME2 2018.4 microbiome bioinformatics platform [29]. QIIME2 was integrated into a non-public instance of Galaxy (MedBionode https://galaxy.medunigraz.at) [30]. Taxonomic assignment of the DADA2 representative sequences was achieved using the QIIME2 sklearn-based classifier for the SILVA rRNA database release 132 at 99% identity [31]. Alpha (inverse Simpson, Chao1, and Shannon) and beta (Bray–Curtis) diversity metrics were computed using RStudio version 2022.12.0 + 353. Relative abundances at various taxonomic levels were extracted and used for group comparisons. Discriminatory biomarkers were identified using LEfSE.

### 2.11. Statistical Analysis

The data were initially managed using Microsoft Excel 2016. Statistical analysis and graphical representation were performed using GraphPad Prism 9 and MetaboAnalyst 5.0 [32]. Metric data are presented as mean and standard deviation (SD). Due to the sample size and absence of normal distribution in most parameters (tested with the Kolmogorov–Smirnov test), group differences between sham and SBS animals were assessed using the Mann–Whitney U-Test. Correlations between significantly different BAs, VOCs and microbiota were calculated using Spearman’s correlation coefficient. A *p*-value of <0.05 was considered statistically significant.

## 3. Results

### 3.1. Operation, Overall Survival and Weight Changes

Of the 22 animals, *n* = 14 were assigned to the SBS groups and *n* = 8 to the sham group. Preoperatively, the mean weights of the animals of the two groups were not significantly different (sham 27.7 ± 2.4 g vs. SBS 28.3 ± 2.6 g; *p* = 0.840). Likewise, the duration of the operative procedures did not significantly differ between the two groups (sham 32 ± 5 min vs. SBS 36 ± 3 min; *p* = 0.069). We observed a 100% survival rate of the sham-operated animals. In contrast, the SBS group exhibited an overall survival rate of 65%, leaving nine animals in this group for further analyses. However, no intraoperative deaths were recorded.

**Figure 1:**
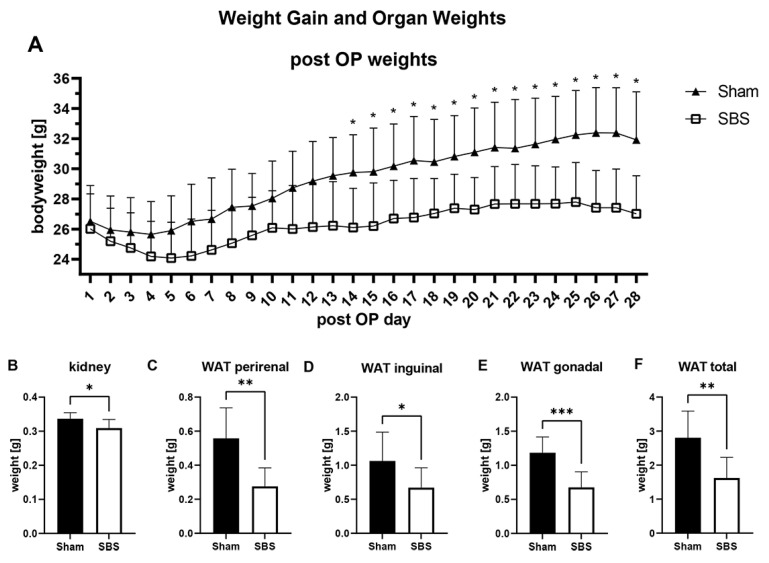
Postoperative weight gain in both groups (**A**); significant reduction in kidney weight (**B**) and significant reduction in white adipose tissue (WAT) in the SBS animals (**C**–**F**); * *p* < 0.05; ** *p* < 0.01; *** *p* < 0.001.

In both groups, we observed postoperative weight loss. However, from postoperative day 14, significantly reduced weights were seen in the SBS group (Figure 1A). At euthanasia four weeks postoperatively, significant reductions in white adipose tissue (perirenal, inguinal, gonadal and total) and a significant reduction in kidney weight were observed in the SBS group (Figure 1B–F). The remaining organ weights (liver, spleen, pancreas) did not significantly differ between the two groups (Supplementary Table S1).

### 3.2. Gut Permeability, Cytokines, Chemokines and Tight Junction Proteins

FITC analysis did not reveal any significant differences between the two groups (sham 0.349 ± 0.078 µL/mL vs. SBS 0.362 ± 0.114 µL/mL; *p* = 0.963). Citrulline and membrane proteins occludin and claudin 2 were not significantly different between the two groups (Table 1). Chemokine/cytokine profiling of 18 substances revealed significant differences in IL-1beta, IL-2R and TNF-α (Table 1).

### 3.3. Short-Chain Fatty Acids (SCFAs)

None of the five measured SFCAs were significantly different between the two groups (acetic acid, propionic acid, butyric acid, pentanoic acid and hexanoic acid). Detailed values are presented in Supplementary Table S2.

### 3.4. Bile Acids in Serum and Stool

While SBS caused a significant increase in quantitative serum BAs (sham 1.227 ± 0.692 µmol/L vs. SBS 10.42 ± 13.24 µmol/L; *p* = 0.015), the total sum of quantitative stool BAs was not significantly different between the groups (sham 25.55 ± 5.761 µmol/g vs. SBS 17.10 ± 11.92 µmol/g; *p* = 0.139). Out of the 15 serum BAs analyzed with a quantitative method, two were not detectable (lithocholic acid, taurolithocholic acid), ten were not significantly different between the two groups and three (deoxycholic acid, taurochenodeoxycholic acid, taurocholic acid) were significantly higher in SBS animals. In the stool samples, all of these 15 analyzed BAs did not significantly differ between sham and SBS animals. Only lithocholic acid showed a tendency (*p* = 0.075) towards a decrease in SBS animals. Detailed values are shown in Table 2.

**Figure 2:**
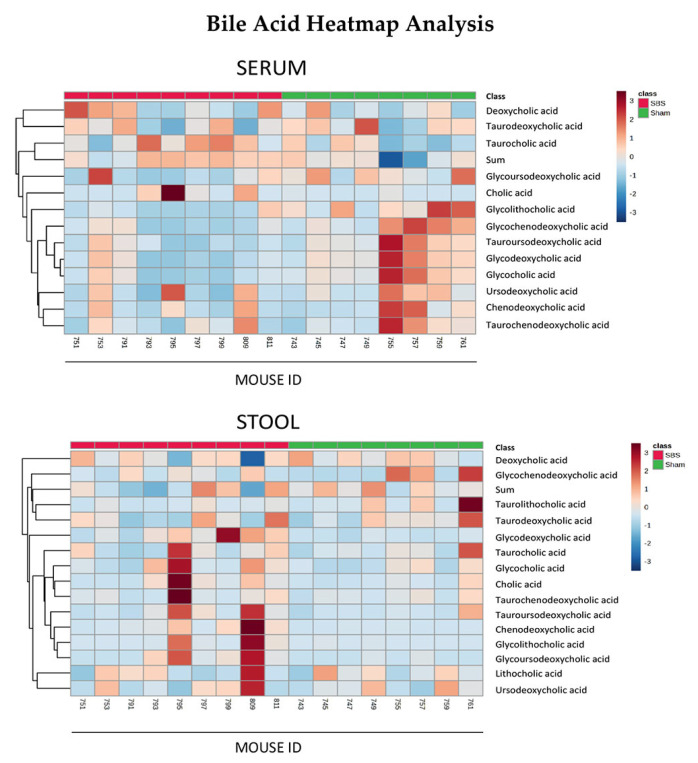
Dendogram heatmap analyses of 15 quantitatively measured BAs in sham and SBS animals in serum and stool.

Dendogram heatmap analyses of the quantitatively analyzed BAs are shown in Figure 2.

Using the extended and qualitative method, in serum samples, 52 BAs were found; 29 were not significantly different between the two groups, 21 were significantly higher in SBS animals and 2 were significantly lower in SBS animals. Applying the same methods in stool samples, 43 BAs were found; 32 were not significantly different between the two groups, 10 were significantly higher in SBS animals and 1 was significantly lower in SBS animals. Nine BAs detected in serum samples could not be found in stool samples. The detailed values are shown in Supplementary Table S3.

### 3.5. Volatile Organic Compounds (VOCs)

**Figure 3:**
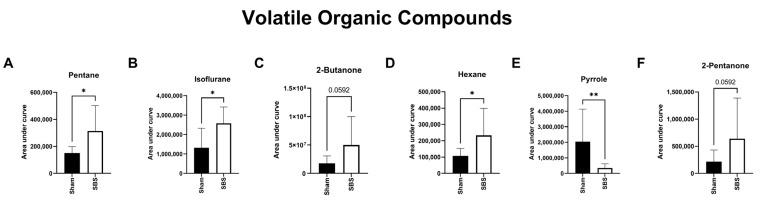
Area under the curve (AUC) of the VOCs in the stool headspace comparing sham (*n* = 8) and SBS animals (*n* = 9); * *p* < 0.05; ** *p* < 0.01. (**A**) penate. (**B**) isofluarne, (**C**) 1-butanone, (**D**) hexane, (**E**) pyrrole, (**F**) 2-pentanone.

In the stool headspace, 36 VOCs could be detected and were used for group comparison. Alterations in 7 substances were significantly affected by room air contamination (levels in room air > 30% of exhaled concentration), leaving 29 substances for further analysis. Out of these, pentane, isoflurane and hexane were significantly increased and pyrrole significantly decreased in SBS animals (Figure 3). 2-Butanone and 2-pentanone showed a clear trend towards increased values in SBS animals. Detailed values are shown in Supplementary Table S4.

### 3.6. Microbiome Analysis

Due to the different read count per sample, rarefication was carried out, and samples with less than 99 reads in the jejunum, 199 reads in the ileum, 3163 reads in the colon and 2491 reads in the stool were excluded from alpha diversity estimations. This concerns one sample of the sham group (colon and jejunum sections) as well as four stool samples (two sham and two SB). Furthermore, no samples of the different intestinal sections could be obtained from one sham mouse due to insufficient quantities. For the total list of reads, see Supplementary Table S5.

**Figure 4:**
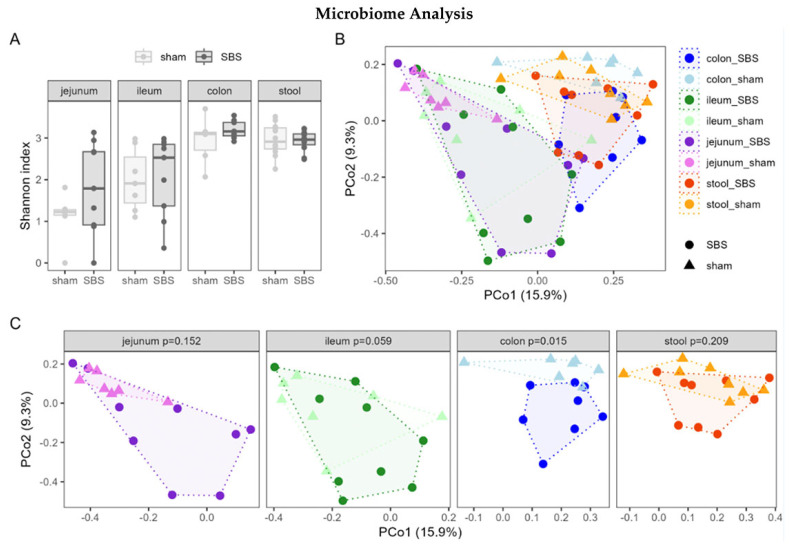
Alpha diversity (**A**) and beta diversity (**B**); PCoA plots for Bray–Curtis dissimilarities, permutational multivariate analysis of variance (PERMANOVA): significant difference between SBS and sham mice (*p* = 0.002) and between the different segments of the intestine (*p* = 0.001); (**C**) PERMANOVA for different segments of the intestine: significant difference in the colon between the two groups.

The microbiome analysis revealed that SBS did not lead to significant alteration of the α-diversity in the jejunum, ileum, colon or stool as assessed by the Shannon index (Figure 4A), observed species, inverse Simpson index or evenness. Beta diversity showed significant differences in the Bray–Curtis dissimilarity among the two groups and different segments of the intestine, as seen in Figure 4B. The graph illustrates cluster formation between colon samples, with significant differences observed between the two groups (Figure 4C).

**Figure 5:**
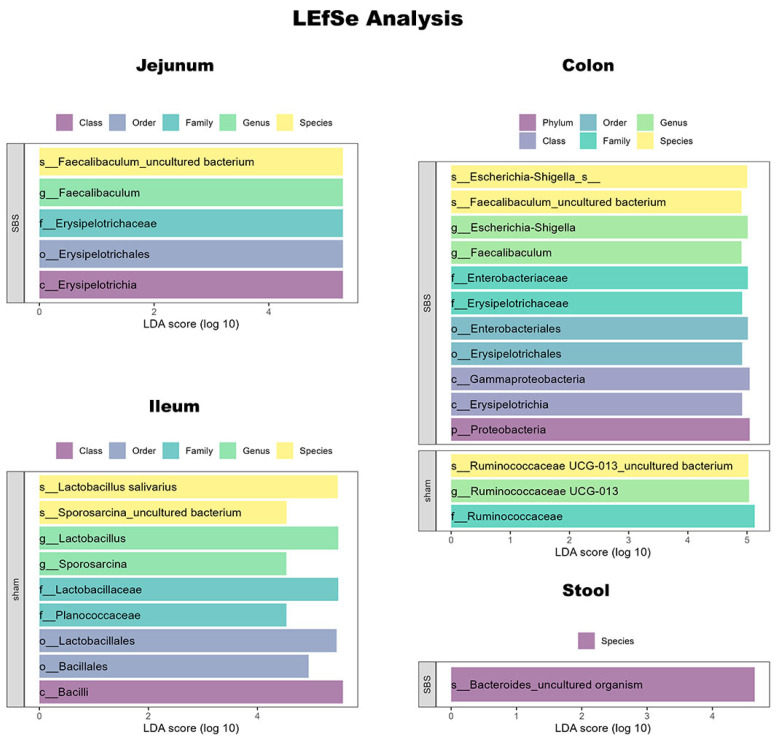
LEfSe analysis comparing sham and SBS animals.

At the species level, the linear discriminant analysis effect size (LEfSe) analysis revealed differences between the two groups (Figure 5). Cladograms of the different sections are depicted in Supplementary Figure S1.

In the colon of SBS mice, *Escherichia-Shigella* and an *uncultured Faecalibacluum* species as well as their respective parent taxa were significantly enriched, while a species of the *Ruminococcaceae UCG-013* genus and the respective family were decreased. *Faecalibaculum* was also enriched in the jejunum of SBS mice. In contrast, the sham mice showed significant enrichment of *Lactobacillus salivarius* and an *uncultured Sporosarcina* species in the ileum. In the stool samples of SBS mice, only an *uncultured Bacteroides* species was significantly enriched.

### 3.7. Correlations

**Figure 6:**
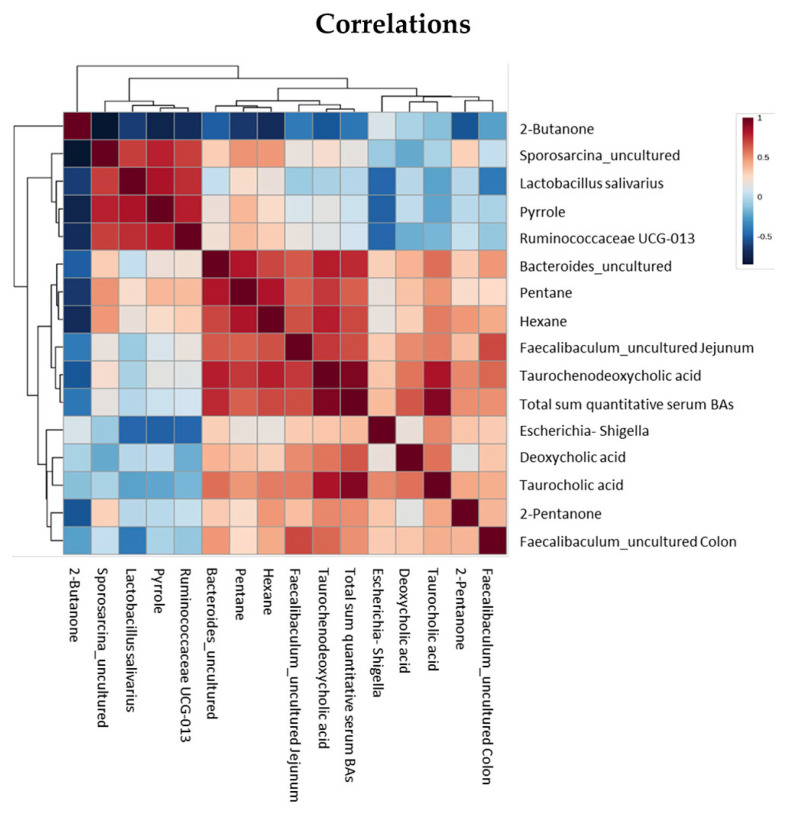
Correlations between significantly different serum BAs, VOCs and microbiota.

Correlation analysis of the significantly different serum BAs, VOCs and microbiota revealed a significant positive correlation of the fecal VOC pyrrole with *Ruminococcaceae UCG-013* (correlation coefficient 0.79, *p* < 0.05), *Lactobacillus salivarius* (correlation coefficient 0.83, *p* < 0.05) and *Sporosarcina_uncultured* (correlation coefficient 0.78, *p* < 0.05) and a significant negative correlation with *Escherichi-Shigella* (correlation coefficient −0.49, *p* < 0.05). Both pentane and hexane were significantly correlated positively with Bacteroides (correlation coefficient 0.82 and 0.69, both *p* < 0.05) and jejunal *Faecalibaculum_uncultured* (correlation coefficient 0.63 and 0.67, both *p* < 0.05). A correlation heatmap is shown in Figure 6. The exact correlation coefficients are displayed in Supplementary Table S6.

## 4. Discussion

The main findings of our study were that four weeks of SBS in mice causes shifts in the intestinal microbiome and metabolome (BAs and VOCs). While alterations in the microbiome and BAs are already known in both clinical and experimental settings, fecal VOCs have not been studied in a murine model yet.

The creation of SBS caused a significant reduction in body weight driven by a reduction in white adipose tissue. Unchanged FITC serum values, membrane proteins and inflammatory markers in serum samples did not suggest a complete breakdown of the intestinal barrier. In a piglet model of SBS, Lapthorne et al. found that inflammatory gene expression in the colon significantly increases six weeks but not two weeks post-surgery [33]. Therefore, analysis of the inflammatory markers at later time points would be of interest for future studies. 

A main finding of our study was that SBS caused alterations in BAs—as assessed by quantitative and qualitative methods—in both stool and serum. BAs represent potent metabolic and immune signaling molecules synthesized in the liver and then transported to the intestine where they undergo metabolism by gut bacteria [34]. Therefore, there is a close relationship between BA metabolism and the intestinal microbiome. Also, alterations in the microbial composition regulate elements of BA metabolism and signaling [15]. Using a quantitative approach, we were able to detect significant increases in taurochenodeoxycholic acid, taurocholic acid and deoxycholic acid and an almost 10-fold increase in BAs in serum samples of SBS animals. In a comprehensive mapping of the gut–liver axis in a piglet model of SBS by Pereira-Fantini and colleagues, the authors found that unconjugated primary BAs were increased in SBS at six weeks post-surgery. Moreover, in stool samples, there were no differences in primary and secondary BAs [35]. Despite certain differences, these findings basically support our data. In our SBS group, the significantly elevated levels of the sum of serum BAs were driven by increases in primary BAs (compare Table 2). In a clinical setting, children with SBS have a predominately primary BA profile associated with a reduction in the diversity and number of BA-transforming bacteria [12,36]. The complex interplay between intestinal bacteria and BA metabolism in SBS is also shown by findings that SBS caused a colonic reduction in *Ruminococcaceae*, with a known role in BA metabolism, in our animals [35,37].

VOCs are chemicals that are—amongst others—emitted from feces and contribute to their odor [38,39]. In the intestine, these substances are generated by metabolic processes involving the epithelium and the microbiota and are also influenced by diet. Studies have shown that fecal VOC profiles differ between patients with or without gastrointestinal diseases such as *Campylobacter* infection, *Clostridium difficile* infection, ulcerative colitis, necrotizing enterocolitis and cholera [40,41,42]. However, regarding SBS, potential alterations in fecal VOCs have only been studied in a very limited number of reports. Budinska and coworkers assessed the fecal VOC profile in 29 adult patients with SBS and found that the fecal concentration of 156 VOCs was significantly different between SBS patients and healthy controls [11]. However, only 14 VOCs contributed by >1% to the total profile. In our experimental setting, four VOCs significantly differed between the two groups, and two showed a clear trend (compare Figure 3). 

Pyrrole was the only VOC that we found to be significantly decreased in the fecal headspace of SBS animals. The substance was first comprehensively studied in 1938 [43]. Pyrroles are characterized by a ring structure composed of four carbon atoms and one nitrogen atom. The pyrrole ring system is found as a chemical subunit in the amino acids proline and hydroxyproline and in colored natural products such as heme and bile pigments. It can be detected in the urine and was associated with psychiatric disorders including anxiety and depression [44]. Pyrrole has also been detected in the stool headspace of healthy controls and patients with *Clostridium difficile* infection, *Campylobacter jejuni* infection and ulcerative colitis [40]. The significant positive correlation between pyrrole and Rumicoccoceae, Lactobacillus and Sporosarcina can serve as a focus for future studies since pyrrole remains a poorly researched biomarker with unclear associations concerning the role of this substance in SBS patients.

Both pentane and hexane are short-chain alkanes and were found to be significantly increased in our animals with SBS. Short-chain hydrocarbons such as pentane and hexane have been reported as biomarkers for oxidative stress [45]. Both of these VOCs have not been studied in the setting of SBS. Several studies performed in inflammatory bowel diseases, however, have suggested the potential role of single-breath-exhaled pentane and hexane as biomarkers [46,47]. For instance, pentane has been found to be increased in patients with active Crohn’s disease and ulcerative colitis compared to controls [47]. As a result of lipid peroxidation, breath pentane has been shown to be positively correlated with the presence of intestinal inflammation [48]. Therefore, the increased values found in the SBS mice in our study might be a reflection of increased intestinal inflammation which is commonly found in this setting [49,50]. Isoflurane is not an endogenously produced compound but is taken up during previous anesthesia. Due to the lipophilic character, isoflurane is distributed among different body compartments. The measured differences in exhaled isoflurane between the groups may indicate a modified compartmental distribution between the groups—e.g., related to lipid compartments—as the duration of the operation and presumably the administered levels of isoflurane were comparable between the two groups.

Taken together, these findings indicate that experimental SBS leads to alterations in VOCs in the fecal headspace. Nevertheless, the question of whether the novel non-invasive metabolomic approach represented by these biomarkers has value regarding SBS still has to be answered. More studies that investigate fecal VOCs and their metabolism are therefore needed in both experimental and clinical SBS.

The intestinal microbiome plays a pivotal role in the function, development and integrity of the intestinal epithelium. As a consequence, alterations in the microbiome associated with SBS have already been studied in a variety of experimental models as well as pediatric and adult patients [15,51,52,53]. However, clinical studies mainly concentrate on analysis of the fecal microbiome, because reliable access to intestinal content in other portions of the small and large bowel seems almost impossible [52,53]. In a study including fecal samples of eight surgical intestinal failure pediatric patients, for instance, significantly decreased α-diversity was found compared to healthy controls [54]. Moreover, the microbiota community structure (beta diversity) of IF patients was found to be distinct from that of healthy controls [54]. However, it is well known that the fecal microbiome does not reflect the microbiome found in the other intestinal segments [55]. Additionally, much of the intestinal pathology associated with SBS such as SIBO occurs in the small bowel which is notoriously difficult to access for both diagnostic and therapeutic purposes [56]. Therefore, it is of importance to study the microbial composition of the different intestinal segments. In pediatric SBS patients with enterostomies, effluents from the proximal intestinal segments are available for studying the microbial composition of more proximal intestinal segments. Zhang and colleagues, for instance, have demonstrated higher bacterial richness in both the stoma effluent samples obtained from SBS patients and fecal samples of controls than in the fecal samples of SBS patients [57]. Likewise, the authors describe significantly different beta diversity between stoma effluents and fecal samples in SBS patients. Moreover, certain bacteria, including Bacteroidetes on the phylum level and *Bifidobacterium*, *Klebsiella*, *Lactobacillus* and *Acinetobacter* on the genus level, were significantly different in a comparison of stoma effluents and feces of SBS patients [57]. Sommovilla and coworkers performed a 50% small bowel resection in mice; analyzed the microbiome of the different sections of the intestine; and found no significant differences in the diversity scores of stool, cecal and ileal contents when comparing SBS to sham-operated mice [13]. The results of our study confirm these findings with no differences in diversity scores between sham and SBS animals in the different examined sections of the intestine. However, while the above-mentioned study found significant differences in the beta diversity only in the ileal contents at 90 days post-resection, our study revealed differences in the microbial composition in the jejunum, ileum, colon and stool of sham and SBS animals. Future studies should examine the importance of these differences and could also focus on previous findings that the luminal (transient) and mucosal (resident) microbiome differs within the intestine [55].

SBS is a rare disease with a variety of different underlying causes. A major factor influencing its outcome is the absence or presence of the ileocecal valve [58,59]. Berlin and coworkers examined microbial changes of stool samples in a comparison of mice undergoing 50% small bowel resection leaving the ileocecal junction intact (comparable to our method), limited ileocecal resection and extended ileocecal resection [60]. Alpha diversity as assessed by the Shannon index remained stable following 50% small bowel resection at postoperative day 5 and was profoundly decreased following the methods associated with resection of the ileocecal valve. Likewise, it has been reported that ileocecal resection causes dramatically more severe intestinal dysbiosis than small bowel resection only in SBS rat models [61]. These findings are comparable to our findings and emphasize the importance of the presence of the ileocecal valve in SBS and are in line with the fact that reanastomosis of comparably short segments of the small bowel but with intact ileocolonic junction usually converts intestinal failure to intestinal insufficiency in humans [62]. 

Microbial analysis of stool samples collected between postoperative days 2 and 7 in mice undergoing small bowel resection has revealed a reduction in alpha diversity and an increase in Proteobacteria; pathobionts such as *Clostridia*, *Shigella* and *Enterococcus* increased after small bowel resection while *Muribaculaceae*, *Lactobacillus* and *Lachnospiraceae* decreased [60]. In our study, the most profound alterations were found in the colon, with SBS causing increases in *Faecalibaculum* and *Escherichia-Shigella* and decreases in *Ruminococcaceae*. In ileal samples, *Lactobacillus salivarius* and *Sporosarcina* were more abundant in sham animals. Saeed and coworkers highlighted the importance of the intestinal microbiome in various diseases in childhood. Reduced abundances of *Lactobacillus* were recorded in diseases such as necrotizing enterocolitis or infantile colic. In contrast, there were increased rates of pathogenic bacteria including *Escherichia coli* in chronic inflammatory bowel diseases [63]. 

Interestingly, the fecal samples were only different concerning an uncultured organism of Bacteroides. Our findings reflect the above-mentioned fact that the microbiota found in fecal samples does not necessarily reflect alterations in the other intestinal segments. 

Taken together, the creation of SBS with the ileocecal valve in continuity causes alterations in the microbiome in the different sections of the intestine. More studies are, however, needed to examine whether the microbiota changes remain stable over time and to assess whether treatment with, for instance, probiotics or postbiotics can sustainably improve these resection-induced microbial alterations. 

The limitations of the present study include that data derived from experimental murine studies cannot necessarily be related to humans. Therefore, an analysis of fecal and potentially breath-exhaled VOCs in pediatric patients should be performed in future studies. Due to rarefaction, we also were not able to report the microbial alterations in all animals undergoing SBS creation or sham operation. Moreover, we present only one time point of four weeks post-resection. Therefore, we are unable to answer the question about the time course of the alteration we found. Nevertheless, our study includes a thorough analysis of the metabolic and microbial consequences of SBS including an analysis of fecal VOCs in an experimental setting for the first time.

## 5. Conclusions

SBS causes shifts in the murine microbiota in all different sections of the intestinal tract. Moreover, the metabolome including serum and fecal BAs and fecal VOCs was significantly different between sham and SBS animals. Future studies are, however, necessary to shed more light on the value of fecal VOCs in both murine models and human patients. 

## Figures and Tables

**Table 1:** Citrulline, membrane proteins and chemokine/cytokine profiling of serum samples of sham (*n* = 8) and SBS animals (*n* = 9).

Citrulline, Membrane Proteins, Chemokine/Cytokines and Tight Junction Proteins			
	Sham	SBS	*p*-Value
Citrulline (ng/mL)	254.0 ± 205.7	339.1 ± 142.8	0.370
Occludin (ng/mL)	8.508 ± 3.383	10.710 ± 3.483	0.167
Claudin 2 (ng/mL)	12.43 ± 2.913	14.03 ± 3.406	0.370
ENA-78 (pg/mL)	1149.0 ± 572.7	1492 ± 634.8	0.423
G-CSF (pg/mL)	12.74 ± 6.885	10.21 ± 2.782	0.815
GM-CSF (pg/mL)	24.23 ± 5.605	19.37 ± 6.924	0.276
IFN beta (pg/mL)	61.95 ± 18.93	43.35 ± 20.52	0.114
IFN gamma (pg/mL)	1.666 ± 0.627	1.220 ± 0.562	0.191
**IL-1 beta (pg/mL)**	**1.890 ± 1.902**	**0.641 ± 0.859**	**0.043**
IL-10 (pg/mL)	13.09 ± 7.802	11.74 ± 7.452	0.943
IL-17F (pg/mL)	16.42 ± 5.279	11.75 ± 5.593	0.167
IL-2 (pg/mL)	10.72 ± 4.825	9.227 ± 5.457	0.743
**IL-2R (pg/mL)**	**39.10 ± 19.30**	**62.42 ± 19.31**	**0.036**
IL-6 (pg/mL)	65.97 ± 22.74	46.97 ± 25.71	0.200
M-CSF (pg/mL)	1.449 ± 0.516	1.056 ± 0.518	0.146
MCP-1 (pg/mL)	28.11 ± 8.401	21.77 ± 7.265	0.139
MCP-5 (pg/mL)	4.740 ± 2.892	3.065 ± 2.188	0.167
MIP-1 alpha (pg/mL)	0.419 ± 0.323	0.420 ± 0.389	0.586
MIP-1 beta (pg/mL)	1.808 ± 0.574	2.158 ± 0.713	0.384
**TNF alpha (pg/mL)**	**10.91 ± 3.670**	**5.403 ± 4.020**	**0.014**
CRP (µg/mL)	133.3 ± 68.67	134.4 ± 43.75	0.673

Data are displayed as means ± SD; significant differences are displayed in bold.

**Table 2:** Fifteen quantitatively measured BAs in sham and SBS animals in serum and stool.

Bile Acids				
	Type	Serum Quantitative		
		Sham	SBS	*p*-Value
Chenodeoxycholic acid	primary	0.0177 ± 0.0100	0.1164 ± 0.2268	0.0927
Cholic acid	primary	0.0126 ± 0.0227	2.1760 ± 4.8080	0.0993
Tauroursodeoxycholic acid	primary	0.0000 ± 0.0000	0.1793 ± 0.3470	0.2059
**Taurochenodeoxycholic acid**	primary	**0.0000 ± 0.0000**	**0.0765 ± 0.0944**	**0.0294**
**Taurocholic acid**	primary	**0.3905 ± 0.4495**	**5.9760 ± 8.8150**	**0.0360**
Glycochenodeoxycholic acid	primary	0.0371 ± 0.0135	0.0343 ± 0.0068	0.9626
**Sum primary BAs**	**primary**	**0.4578 ± 0.4527**	**8.559 ± 12.28**	**0.0464**
Lithocholic acid	secondary	0.0000 ± 0.0000	0.0000 ± 0.0000	1.0000
**Deoxycholic acid**	secondary	**0.1292 ± 0.1295**	**0.5447 ± 0.3098**	**0.0080**
Glycolithocholic acid	secondary	0.1082 ± 0.1157	0.0241 ± 0.0721	0.0905
Glycoursodeoxycholic acid	secondary	0.0686 ± 0.0734	0.1064 ± 0.0603	0.7517
Glycocholic acid	secondary	0.1105 ± 0.0140	0.1438 ± 0.0620	0.2359
Taurolithocholic acid	secondary	0.0000 ± 0.0000	0.0000 ± 0.0000	1.0000
Taurodeoxycholic acid	secondary	0.1322 ± 0.1089	0.4403 ± 0.2887	0.3334
Glycodeoxycholic acid	secondary	0.1703 ± 0.0023	0.1711 ± 0.0025	0.6058
**Sum secondary BAs**	**secondary**	**0.719 ± 0.2963**	**1.43 ± 0.838**	**0.0464**
Ursodeoxycholic acid	tertiary	0.0510 ± 0.0107	0.4350 ± 1.0120	0.0592
**Total Sum**		**1.2270 ± 0.6919**	**10.4200 ± 13.2400**	**0.0152**
	**Type**	**stool quantitative**		
		**sham**	**SBS**	***p*-value**
Chenodeoxycholic acid	primary	0.0193 ± 0.0052	0.0371 ± 0.0423	0.2359
Cholic acid	primary	0.2823 ± 0.3232	0.2876 ± 0.2570	0.8148
Tauroursodeoxycholic acid	primary	0.0440 ± 0.0423	0.0317 ± 0.0260	0.1996
Taurochenodeoxycholic acid	primary	0.0185 ± 0.0112	0.0125 ± 0.0072	0.3213
Taurocholic acid	primary	0.4305 ± 0.3866	0.2554 ± 0.2598	0.1672
Glycochenodeoxycholic acid	primary	0.0003 ± 0.0005	0.0001 ± 0.0002	0.5629
Sum primary BAs	primary	0.7949 ± 0.7529	0.6244 ± 0.4628	0.5414
Lithocholic acid	secondary	2.0650 ± 1.4540	0.9991 ± 0.7162	0.0745
Deoxycholic acid	secondary	21.3900 ± 4.2740	14.5700 ± 10.5800	0.2359
Glycolithocholic acid	secondary	0.0004 ± 0.0007	0.0006 ± 0.0007	0.4923
Glycoursodeoxycholic acid	secondary	0.0004 ± 0.0008	0.0013 ± 0.001	0.1332
Glycocholic acid	secondary	0.0041 ± 0.0032	0.0032 ± 0.0020	0.8884
Taurolithocholic acid	secondary	0.0013 ± 0.0021	0.0003 ± 0.0004	0.2176
Taurodeoxycholic acid	secondary	0.1080 ± 0.0774	0.0791 ± 0.0796	0.4234
Glycodeoxycholic acid	secondary	0.0060 ± 0.0027	0.0122 ± 0.0209	0.9626
Sum secondary BAs	secondary	23.71 ± 5.354	15.66 ± 11.01	0.1672
Ursodeoxycholic acid	tertiary	1.1780 ± 0.6763	0.8077 ± 0.7047	0.1996
Total Sum		25.5500 ± 5.7610	17.1000 ± 11.9200	0.1388

Data are displayed as means ± SD; significant differences are displayed in bold.

## Data Availability

The datasets are available from the corresponding author upon reasonable request.

## Supplementary Materials

The following supporting information can be downloaded at: https://www.mdpi.com/article/10.3390/nu15234949/s1, Supplementary Figure S1: Microbiome analysis: cladograms of different sections; Supplementary Table S1: Organ weights; Supplementary Table S2: SCFA analysis; Supplementary Table S3: Qualitatively measured BAs in serum and stool; Supplementary Table S4: Integrated areas of the VOCs; Supplementary Table S5: Reads microbiome analysis; Supplementary Table S6: Correlation coefficients.
