# Supplementary material for: Metabolomic Alterations of Volatile Organic Compounds and Bile Acids as Biomarkers of Microbial Shifts in a Murine Model of Short Bowel Syndrome
Source: Nutrients. 2023 Nov 29;15(23):4949. doi: 10.3390/nu15234949 (PMC10708115; doi:10.3390/nu15234949)
Supplement: Supplementary file 1 [file nutrients-15-04949-s001.zip › Supplementary Table S1.docx]

**Supplementary Table S1**: Weight of liver, spleen and pancreas of sham (n=8) and SBS animals (n=9).

|  | **sham** | **SBS** | **p-value** |
| --- | --- | --- | --- |
| liver [g] | 1.492 ± 0.191 | 1.394 ± 0.254 | 1 |
| spleen [g] | 0.057 ± 0.005 | 0.06 ± 0.01 | 0.541 |
| pancreas [g] | 0.220 ± 0.030 | 0.211 ± 0.041 | 0.606 |
