# Supplementary material for: Metabolomic Alterations of Volatile Organic Compounds and Bile Acids as Biomarkers of Microbial Shifts in a Murine Model of Short Bowel Syndrome
Source: Nutrients. 2023 Nov 29;15(23):4949. doi: 10.3390/nu15234949 (PMC10708115; doi:10.3390/nu15234949)
Supplement: Supplementary file 1 [file nutrients-15-04949-s001.zip › Supplementary Table S2.docx]

**Supplementary Table S2**: SCFAs in the cecal content of sham (n=8) and SBS animals (n=9). Values are shown in µmol/kg.

|  | **sham** | **SBS** | **p-value** |
| --- | --- | --- | --- |
| acetic acid | 500.5 ± 288.3 | 543.3 ± 311.9 | 0.815 |
| propionic acid | 97.66 ± 47.85 | 121.2 ± 88.86 | 0.673 |
| butyric acid | 63.90 ± 42.87 | 68.56 ± 44.19 | 0.815 |
| pentanoic acid | 12.71 ± 7.847 | 15.23 ± 11.62 | 0.815 |
| hexaonic acid | 0.188 ± 0.113 | 0.144 ± 0.073 | 0.391 |
