# Supplementary material for: Metabolomic Alterations of Volatile Organic Compounds and Bile Acids as Biomarkers of Microbial Shifts in a Murine Model of Short Bowel Syndrome
Source: Nutrients. 2023 Nov 29;15(23):4949. doi: 10.3390/nu15234949 (PMC10708115; doi:10.3390/nu15234949)
Supplement: Supplementary file 1 [file nutrients-15-04949-s001.zip › Supplementary Table S3.docx]

**SupplementaryTable S3**: Qualitatively measured BAs in sham and SBS animals in serum and stool, statistically significant differences are printed in bold.

|  | **serum qualitative** | | | **stool qualitative** | | |
| --- | --- | --- | --- | --- | --- | --- |
|  | **sham** | **SBS** | **p-value** | **sham** | **SBS** | **p-value** |
| **Ursocholanic acid** | **43075 ± 15194** | **108962 ± 71584** | **0.0025** | **92349 ± 31271** | **53347 ± 22791** | **0.0206** |
| **5-Cholenic acid-3B-ol** | **20148 ± 5956** | **7234 ± 9059** | **0.0142** | **307000 ± 291312** | **84818 ± 63303** | **0.0464** |
| Lithocholenic acid | 1759 ± 2851 | 4020 ± 5252 | 0.3016 | 16051± 4944 | 12973 ± 10020 | 0.2359 |
| **Dehydrolithocholic acid** | **5160 ± 4482** | **13492 ± 8455** | **0.0487** | 8369 ± 10079 | 5982 ± 6198 | 0.9870 |
| **Lithocholic acid** | **32246 ± 22819** | **137890 ± 134290** | **0.0152** | 351469 ± 262813 | 159365 ± 118183 | 0.0927 |
| **23-Nordeoxycholic acid** | **1439 ± 1022** | **10966 ± 20154** | **0.0360** | **29944 ± 44701** | **5920 ± 5242** | **0.0206** |
| **9(11),(5B)-Cholenicacid-3B-ol-12-one** | 3033 ± 4446 | 6494 ± 10048 | 0.9870 | **226424 ± 134036** | **71953 ± 82746** | **0.0055** |
| 3,12-Diketocholanic acid | 2648 ± 2031 | 7213 ± 6875 | 0.1736 | 721463 ± 471705 | 269863 ± 320747 | 0.0592 |
| **3,7-Diketocholanic acid** | **8404 ± 3135** | **3282 ± 3653** | **0.0149** | 50638 ± 36503 | 23474 ± 20550 | 0.0624 |
| **3,6-Diketocholanic acid** | 8801 ± 5916 | 4914 ± 4121 | 0.2346 | **9044 ± 6868** | **3198 ± 1880** | **0.0464** |
| 5a-Cholanic acid-3,6-dione | 3033 ± 4446 | 6494 ± 10048 | 0.9870 | N/A | N/A |  |
| 6-Ketolithocholic acid | 28713 ± 18561 | 20277 ± 14403 | 0.2872 | 7730097±4008380 | 4303438 ± 4356018 | 0.1388 |
| **7-Ketolithocholic acid** | 22252 ± 5033 | 22952 ± 11448 | 0.8148 | **883801 ± 427704** | **357595 ± 317633** | **0.0206** |
| **5a-Cholanic acid-3a-ol-6-one** | 39990 ± 8530 | 34849 ± 6103 | 0.3213 | **795623 ± 419099** | **299626 ± 260410** | **0.0145** |
| 12-Ketolithocholic acid | 1444 ± 1330 | 4813 ± 8893 | 1.0000 | 732918 ± 559418 | 441049 ± 441660 | 0.3213 |
| Apocholic acid | 2301 ± 1658 | 1600 ± 3051 | 0.0749 | 239896 ± 165265 | 119924 ± 116579 | 0.1139 |
| 3-Ketodeoxycholic acid | 1315 ± 1748 | 1817 ± 1781 | 0.3043 | 102252 ± 96890 | 42559 ± 40425 | 0.2359 |
| **3-Ketochenodeoxycholic acid** | 1367 ± 1186 | 3036 ± 1886 | 0.1071 | **489203 ± 217461** | **311036 ± 221756** | **0.0360** |
| Murideoxycholic acid | 4653 ± 3957 | 8053 ± 6046 | 0.2359 | 25835994±8205952 | 17933218±14470052 | 0.0745 |
| **Ursodeoxycholic acid** | **3450 ± 3428** | **101245 ± 242110** | **0.0024** | 1399905 ± 844160 | 967434 ± 994777 | 0.1996 |
| Hyodeoxycholic acid | 797 ± 1044 | 6088 ± 13999 | 0.8667 | 641963 ± 427182 | 275779 ± 215693 | 0.0592 |
| 5B-Cholanic acid-3B,12a-diol | 1294 ± 1759 | 4881 ± 8220 | 0.4054 | 1017580 ± 599088 | 788512 ± 915949 | 0.3704 |
| Chenodeoxycholic acid | 1166 ± 1574 | 26355 ± 52776 | 0.0561 | 25835994±8205952 | 17933218±14470052 | 0.0745 |
| **Deoxycholic acid** | **119758 ± 56657** | **350636 ± 161140** | **0.0079** | 1399905 ± 844160 | 967434 ± 994777 | 0.1996 |
| **7a,12a-Dihydroxy-5B-cholan-24-oic acid** | **2344 ± 4369** | **15620 ± 17848** | **0.0211** | N/A | N/A |  |
| Dehydrocholic acid | 20040 ± 7898 | 20932 ± 5424 | 0.8148 | 902 ± 1079 | 256 ± 327 | 0.3032 |
| 7,12-Diketolithocholic acid | 1609 ± 1323 | 14826 ± 31042 | 0.3143 | 0 ± 0 | 0 ± 0 |  |
| 6,7-Diketolithocholic acid | 447 ± 782 | 1867 ± 4067 | 0.5042 | N/A | N/A |  |
| 12-Ketochenodeoxycholic acid | 6191 ± 4627 | 591663±1462434 | 0.8884 | 129292 ± 117626 | 95697 ± 52648 | 0.8884 |
| 7-Ketodeoxycholic acid | 509 ± 656 | 36805 ± 103726 | 0.2299 | 0 ± 0 | 169 ± 479 |  |
| **Takeda ketol** | **5355 ± 4737** | **128787 ± 295837** | **0.0055** | 826 ± 1162 | 755 ± 962 | 0.7979 |
| **Ursocholic acid** | **33824 ± 19539** | **997294±2029563** | **0.0274** | **4353179 ± 3793064** | **2779888 ± 4271079** | **0.0360** |
| **alpha-Muricholic acid** | **7837 ± 5362** | **626276±1546399** | **0.0025** | 0 ± 0 | 0 ± 0 |  |
| **omega-Muricholic acid** | **7211 ± 7205** | **394568±843327** | **0.0010** | 0 ± 0 | 0 ± 0 |  |
| **beta-Muricholic acid** | **588 ± 608** | **24333 ± 55841** | **0.0080** | 0 ± 0 | 0 ± 0 |  |
| Hyocholic acid | 935 ± 880 | 42972 ± 105114 | 0.0852 | 0 ± 0 | 0 ± 0 |  |
| Cholic acid | 0 ± 0 | 969353±2098892 | 0.0824 | 224368 ± 192322 | 345530 ± 333747 | 0.6058 |
| **Glycoursodeoxycholic acid** | **30269 ± 1124** | **33359 ± 3235** | **0.0334** | 1836 ± 1096 | 4040 ± 7728 | 0.5881 |
| **Glycohyodeoxycholic acid** | **30269 ± 1124** | **33359 ± 3235** | **0.0334** | 340 ± 356 | 286 ± 168 | 0.8703 |
| **Glycochenodeoxycholic acid** | **30512 ± 829** | **33489 ± 3163** | **0.0434** | N/A | N/A |  |
| **Glycodeoxycholic acid** | **30269 ± 1124** | **33359 ± 3235** | **0.0334** | N/A | N/A |  |
| Glycohyocholic acid | 92 ± 130 | 1041 ± 2379 | 0.9015 | 771 ± 498 | 665 ± 448 | 0.6927 |
| Glycocholic acid | 5118 ± 1318 | 8859 ± 7001 | 0.3088 | N/A | N/A |  |
| Tauroursodeoxycholic acid | 31184 ± 17143 | 91941 ± 89161 | 0.1672 | 31792 ± 22005 | 23488 ± 22091 | 0.5414 |
| Taurohyodeoxycholic acid | 3018 ± 1690 | 13634 ± 18696 | 0.2449 | 1661 ± 1318 | 568 ± 543 | 0.0556 |
| **Taurochenodeoxycholic acid** | **567 ± 478** | **4668 ± 4542** | **0.0274** | **5246 ± 2786** | **2276 ± 1151** | **0.0164** |
| Taurodeoxycholic acid | 29424 ± 15467 | 77672 ± 85790 | 0.5414 | 5866 ± 4886 | 2947 ± 3118 | 0.1388 |
| **Tauro-alpha-muricholic acid** | **4401 ± 3323** | **80960 ± 92001** | **0.0035** | 72844 ± 40108 | 41490 ± 34313 | 0.1388 |
| Tauro-omega-muricholic acid | 3348 ± 2780 | 33542 ± 42814 | 0.0592 | N/A | N/A |  |
| **Tauro-beta-muricholic acid** | **8368 ± 7644** | **172973 ± 246935** | **0.0360** | N/A | N/A |  |
| **Taurohyocholic acid** | **214 ± 176** | **9926 ± 16339** | **0.0034** | N/A | N/A |  |
| **Taurocholic acid** | **39124 ± 32744** | **410072 ± 440204** | **0.0360** | **42425 ± 18764** | **318953 ± 396759** | **0.0360** |
