# Supplementary material for: Metabolomic Alterations of Volatile Organic Compounds and Bile Acids as Biomarkers of Microbial Shifts in a Murine Model of Short Bowel Syndrome
Source: Nutrients. 2023 Nov 29;15(23):4949. doi: 10.3390/nu15234949 (PMC10708115; doi:10.3390/nu15234949)
Supplement: Supplementary file 1 [file nutrients-15-04949-s001.zip › Supplementary Table S4.docx]

**Supplementary Table S4**: Integrated areas of the VOCs analyzed in the fecal headspace of sham (n=8) and SBS animals (n=9). Significantly different VOCs are shown in bold.

|  | **Sham [area under curve] ± SD** | **SBS [area under curve] ± SD** | **p-value** |
| --- | --- | --- | --- |
| Acetaldehyde | 5895766 ± 3959596 | 43344879 ± 2708069 | 0.481 |
| Methanethiol | 40741 ± 31168 | 82911 ± 73213 | 0.139 |
| Acetonitrile | 53991 ± 32442 | 86258 ± 87196 | 0.815 |
| Propanal | 3874168 ± 2626362 | 2896790 ± 1413483 | 0.481 |
| Acetone | 33847493 ± 17705532 | 85944847 ± 100233515 | 0.321 |
| Carbondisulfide | 1024225 ± 981500 | 2025153 ± 1493869 | 0.139 |
| Dimethylsulfide | 46263 ± 39598 | 48348 ± 25837 | 0.541 |
| Acetic Acide ME | 352808 ± 298445 | 253340 ± 163396 | 0.815 |
| **Pentane** | **150767 ± 48474** | **313692 ± 187881** | **0.046** |
| **Isoflurane** | **1325592 ± 1002186** | **2573654 ± 842828** | **0.027** |
| 2-Methylpropanal | 370728 ± 187364 | 571290 ± 594456 | 0.743 |
| Methylvinylketone | 27068 ± 29336 | 72919 ± 114636 | 0.798 |
| 2,3-Butandione | 43145763 ± 448115234 | 17590822 ± 15217756 | 0.236 |
| 2-Butanone | 17658724 ± 13281450 | 50057938 ± 49932860 | 0.059 |
| Ethylacetate | 912125 ± 896563 | 1177226 ± 1584235 | 0.888 |
| Benzene | 1927463 ± 1606373 | 788717 ± 404132 | 0.541 |
| **Hexane** | **107902 ± 45445** | **232766 ± 166073** | **0.046** |
| **Pyrrole** | **2042658 ± 2095106** | **346304 ± 271853** | **0.005** |
| 3-Methylbutanal | 976462 ± 1133842 | 1337709 ± 1829483 | 0.422 |
| 2-Pentanone | 215497 ± 216452 | 638905 ± 750997 | 0.059 |
| 2,3-Pentanedione | 970614 ± 1130730 | 1077809 ± 1119221 | 0.962 |
| Propionic Acid EE | 25658 ± 39133 | 268400 ± 531073 | 0.772 |
| n-Propylacetate | 526741 ± 453285 | 594925 ±830328 | 0.948 |
| Toluene | 604259 ± 298020 | 450871 ± 126844 | 0.481 |
| 2-Hexanone | 58634 ± 69906 | 200648 ± 297568 | 0.317 |
| Hexanal | 153033 ± 82330 | 91386 ± 45087 | 0.114 |
| Octane | 147153 ± 82717 | 255659 ± 221631 | 0.277 |
| Benzaldehyde | 3193936 ± 2378703 | 4729236 ± 4812232 | 0.963 |
| Indole | 5873048 ± 5018756 | 9938988 ± 9002109 | 0.370 |
