# Supplementary material for: Metabolomic Alterations of Volatile Organic Compounds and Bile Acids as Biomarkers of Microbial Shifts in a Murine Model of Short Bowel Syndrome
Source: Nutrients. 2023 Nov 29;15(23):4949. doi: 10.3390/nu15234949 (PMC10708115; doi:10.3390/nu15234949)
Supplement: Supplementary file 1 [file nutrients-15-04949-s001.zip › Supplementary Table S5.docx]

**Supplementary Table S5**: Reads microbiome analysis

|  | **group** | **section** | **reads** | **section** | **reads** | **section** | **reads** | **section** | **reads** |
| --- | --- | --- | --- | --- | --- | --- | --- | --- | --- |
| 751 | SBS | jejunum | 99** | ileum | 199* | colon | 7324 | stool | 3130 |
| 753 | SBS | jejunum | 5932 | ileum | 375 | colon | 4476 | stool | 50** |
| 791 | SBS | jejunum | 368 | ileum | 425 | colon | 4314 | stool | 3322 |
| 793 | SBS | jejunum | 2989 | ileum | 4565 | colon | 5721 | stool | 5549 |
| 795 | SBS | jejunum | 5995 | ileum | 5249 | colon | 5427 | stool | 5124 |
| 797 | SBS | jejunum | 922 | ileum | 4441 | colon | 7953 | stool | 3341 |
| 799 | SBS | jejunum | 132 | ileum | 305 | colon | 4307 | stool | 382** |
| 809 | SBS | jejunum | 4919 | ileum | 5954 | colon | 4475 | stool | 4420 |
| 811 | SBS | jejunum | 1608 | ileum | 3448 | colon | 3952 | stool | 3225 |
| 743 | sham | jejunum | 129 | ileum | 6072 | colon | 772** | stool | 2491* |
| 745 | sham | jejunum | 220 | ileum | 255 | colon | 3522 | stool | 412** |
| 747 | sham | jejunum | 351 | ileum | 715 | colon | 8504 | stool | 5236 |
| 749 | sham | jejunum | 861 | ileum | 2309 | colon | 3279 | stool | 2876 |
| 755 | sham | jejunum | - | ileum | - | colon | - | stool | 59** |
| 757 | sham | jejunum | 22* | ileum | 5581 | colon | 5010 | stool | 4508 |
| 759 | sham | jejunum | 334 | ileum | 1360 | colon | 3163* | stool | 6009 |
| 761 | sham | jejunum | 222 | ileum | 469 | colon | 5050 | stool | 2944 |

* sorted out due to too less read counts; ** lower limit of read counts
