# Supplementary figures and images for: Metabolomic Alterations of Volatile Organic Compounds and Bile Acids as Biomarkers of Microbial Shifts in a Murine Model of Short Bowel Syndrome
Source: Nutrients. 2023 Nov 29;15(23):4949. doi: 10.3390/nu15234949 (PMC10708115; doi:10.3390/nu15234949)

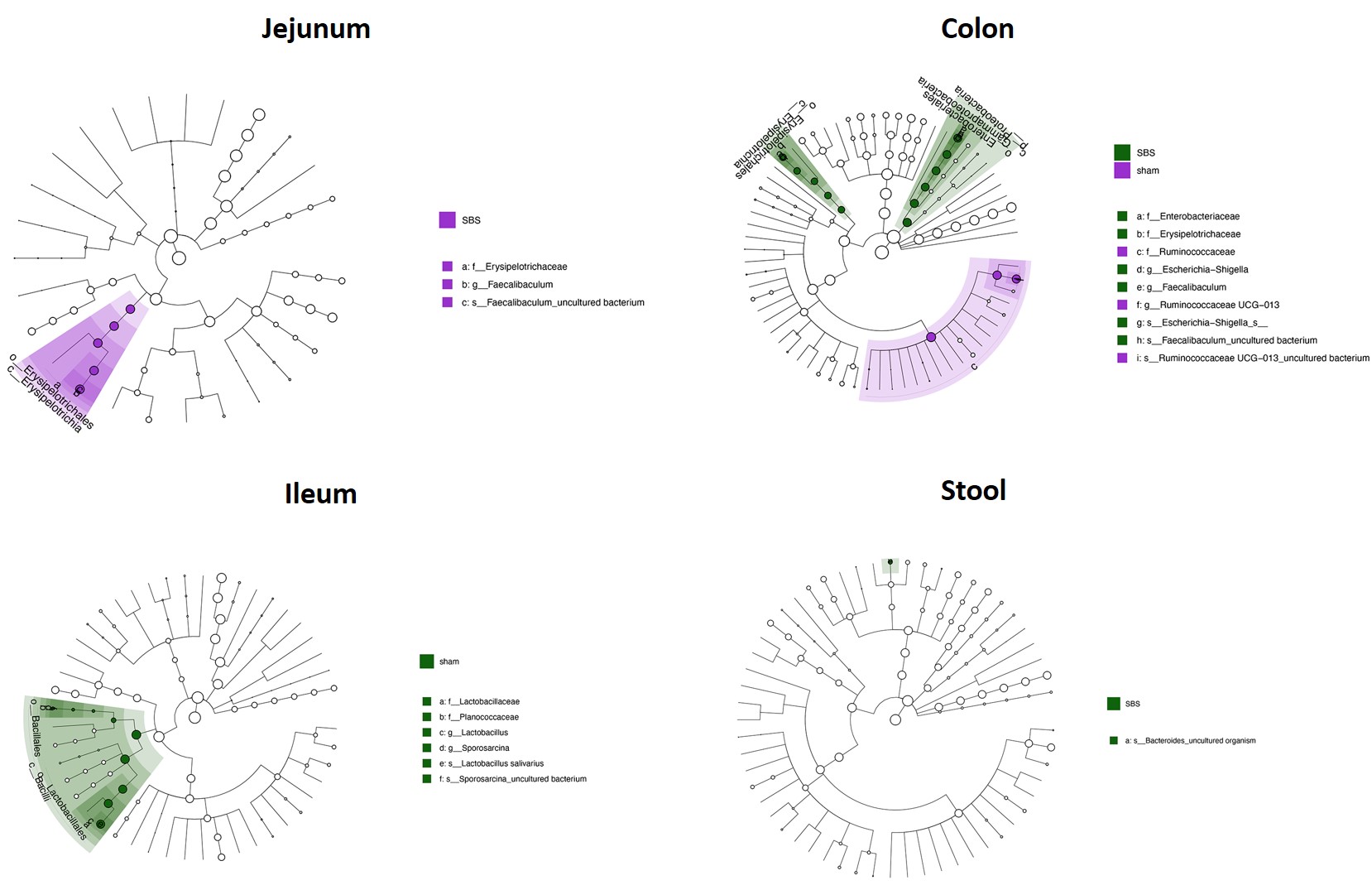

Supplement: Supplementary file 1 [file nutrients-15-04949-s001.zip › Supplementary Figure S1.jpg]
